# 2-Carb­oxy­pyridinium maleate

**DOI:** 10.1107/S1600536812041177

**Published:** 2012-10-06

**Authors:** P. Pandi, G. Peramaiyan, R. Akilan, G. Chakkaravarthi, R. Mohankumar

**Affiliations:** a Department of Physics, Panimalar Engineering College, Chennai 600 123, India; b Department of Physics, Presidency College, Chennai 600 005, India; c Department of Physics, Aksheyaa College of Engineering, Kancheepuram 603 314, India; d Department of Physics, CPCL Polytechnic College, Chennai 600 068, India

## Abstract

In the title mol­ecular salt, C_6_H_6_NO_2_^+^ **^.^**C_4_H_3_O_4_^−^, the 2-carb­oxy­pyridinium cation is essentially planar with a maximum deviation of 0.003 (3) Å. In the crystal, adjacent cations and anions are linked by an extensive system of weak N—H⋯O, O—H⋯O and C—H⋯O inter­actions, forming a layer parallel to the *ab* plane.

## Related literature

For details of pyridine and its derivatives, see: Banerjee & Murugavel (2004 ▶); Bis *et al.* (2006 ▶). For bond-length data, see: Allen *et al.* (1987 ▶).
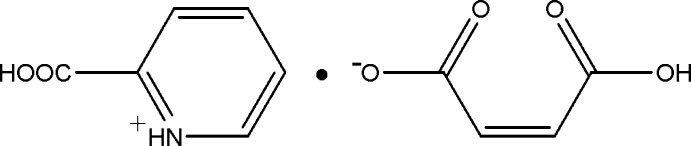


## Experimental

### 

#### Crystal data

C_6_H_6_NO_2_·C_4_H_3_O_4_
*M* *_r_* = 239.18
Monoclinic, 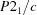

*a* = 14.6498 (9) Å
*b* = 10.3976 (8) Å
*c* = 6.9067 (5) Å
β = 100.089 (3)°
*V* = 1035.78 (13) Å^3^
*Z* = 4
Mo *K*α radiation
μ = 0.13 mm^−1^
*T* = 295 K
0.24 × 0.20 × 0.16 mm

#### Data collection

Bruker Kappa APEXII CCD diffractometer
Absorption correction: multi-scan (*SADABS*; Sheldrick, 1996 ▶) *T*_min_ = 0.970, *T*_max_ = 0.980
9722 measured reflections
2557 independent reflections
2092 reflections with *I* > 2σ(*I*)
*R*_int_ = 0.027

#### Refinement

*R*[*F* ^2^ > 2σ(*F* ^2^)] = 0.071
*wR*(*F* ^2^) = 0.199
*S* = 1.10
2557 reflections
155 parameters
H-atom parameters constrained
Δρ_max_ = 0.43 e Å^−3^
Δρ_min_ = −0.33 e Å^−3^

### 

Data collection: *APEX2* (Bruker, 2004 ▶); cell refinement: *SAINT* (Bruker, 2004 ▶); data reduction: *SAINT*; program(s) used to solve structure: *SHELXS97* (Sheldrick, 2008 ▶); program(s) used to refine structure: *SHELXL97* (Sheldrick, 2008 ▶); molecular graphics: *PLATON* (Spek, 2009 ▶); software used to prepare material for publication: *SHELXL97*.

## Supplementary Material

Crystal structure: contains datablock(s) global, I. DOI: 10.1107/S1600536812041177/rk2380sup1.cif

Structure factors: contains datablock(s) I. DOI: 10.1107/S1600536812041177/rk2380Isup2.hkl

Supplementary material file. DOI: 10.1107/S1600536812041177/rk2380Isup3.cml

Additional supplementary materials:  crystallographic information; 3D view; checkCIF report

## Figures and Tables

**Table 1:** Hydrogen-bond geometry (Å, °)

*D*—H⋯*A*	*D*—H	H⋯*A*	*D*⋯*A*	*D*—H⋯*A*
N1—H1⋯O2	0.86	2.28	2.639 (3)	105
O5—H5*A*⋯O4	0.82	1.73	2.540 (3)	168
N1—H1⋯O1^i^	0.86	2.02	2.725 (3)	139
O2—H2*A*⋯O3^ii^	0.82	1.71	2.463 (3)	152
C2—H2⋯O2^iii^	0.93	2.54	3.462 (4)	170
C3—H3⋯O6^iv^	0.93	2.59	3.221 (4)	125
C5—H5⋯O4^v^	0.93	2.40	3.251 (4)	152
C8—H8⋯O6^vi^	0.93	2.40	3.285 (4)	158

Symmetry codes: (i) 
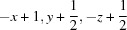
; (ii) 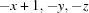; (iii) 
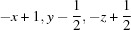
; (iv) 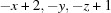; (v) 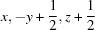; (vi) 
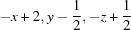
.

**Fig. 1.**
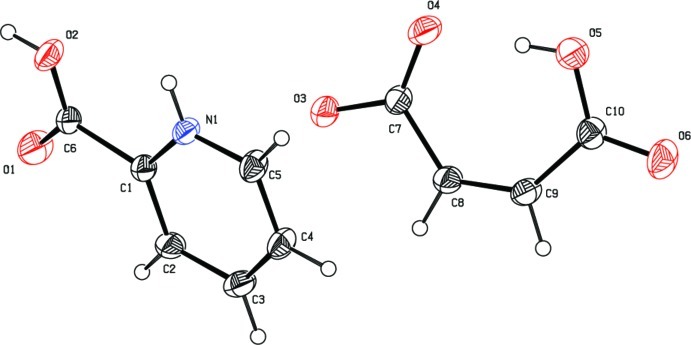
The molecular structure of title compound with atom labels. Displacement ellipsoids are drawn at 30% probability level. H atoms are presented as asmall spheres of arbitrary radius.

**Fig. 2.**
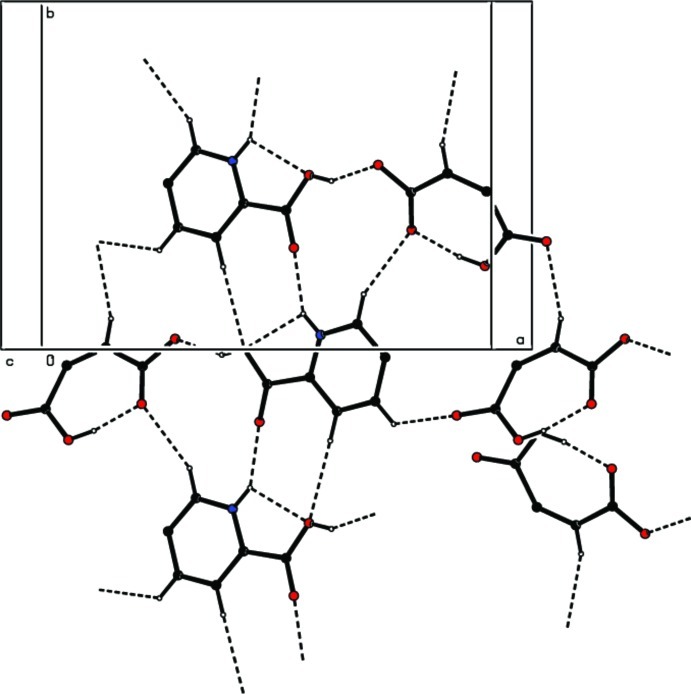
The packing of I, viewed down c axis. Intermolecular hydrogen bonds are shown as dashed lines. H atoms not involved in hydrogen bonding have been omitted for clarity.

## supplementary crystallographic information

### Comment

Pyridine and its derivatives are some of the most frequently used synthons in supramolecular chemistry based on hydrogen bonds (Banerjee & Murugavel, 2004; Bis *et al.*, 2006). We herewith report the molecular and crystal structures of the title compound, I, which belongs to this class of compounds.

The asymmetric unit of I, (Fig. 1), contains a 2–carboxypyridinium cation and a malonate anion. The bond lengths (Allen *et al.*, 1987) and angles are within the normal range. The crystal structure exhibit weak intermolecular N—H···O, O—H···O and C—H···O (Table 1 & Fig. 2) interactions.

### Experimental

A solution of picolinic acid (0.123 g, 1 mmol) in 10 ml ethanol was added with stirring to a solution of maleic acid (0.116 g, 1 mmol) in 10 ml of distilled water at 303 K. After some time, white precipitate was obtained, which was dissolved in ethanol and colourless block–shaped single crystals were obtained by slow evaporation of the ethanol solution.

### Refinement

H atoms were positioned geometrically and refined using riding model with C—H = 0.93Å and *U*_iso_(H) = 1.2*U*_eq_(C) for aromatic H, N—H = 0.86Å and *U*_iso_(H) = 1.2*U*_eq_(N) for amine H and O—H = 0.82Å and *U*_iso_(H) = 1.5*U*_eq_(O) for hydroxyl H.

### Crystal data

**Table:**

C_6_H_6_NO_2_·C_4_H_3_O_4_	*F*(000) = 496
*M**_r_* = 239.18	*D*_x_ = 1.534 Mg m^−^^3^
Monoclinic, *P*2_1_/*c*	Mo *K*α radiation, λ = 0.71073 Å
Hall symbol: -P 2ybc	Cell parameters from 4245 reflections
*a* = 14.6498 (9) Å	θ = 2.4–28.3°
*b* = 10.3976 (8) Å	µ = 0.13 mm^−^^1^
*c* = 6.9067 (5) Å	*T* = 295 K
β = 100.089 (3)°	Block, colourless
*V* = 1035.78 (13) Å^3^	0.24 × 0.20 × 0.16 mm
*Z* = 4	

### Data collection

**Table:**

Bruker Kappa APEXII CCD diffractometer	2557 independent reflections
Radiation source: fine-focus sealed tube	2092 reflections with *I* > 2σ(*I*)
Graphite monochromator	*R*_int_ = 0.027
ω– and φ–scans	θ_max_ = 28.3°, θ_min_ = 1.4°
Absorption correction: multi-scan (*SADABS*; Sheldrick, 1996)	*h* = −19→18
*T*_min_ = 0.970, *T*_max_ = 0.980	*k* = −13→13
9722 measured reflections	*l* = −9→9

### Refinement

**Table:**

Refinement on *F*^2^	Secondary atom site location: difference Fourier map
Least-squares matrix: full	Hydrogen site location: inferred from neighbouring sites
*R*[*F*^2^ > 2σ(*F*^2^)] = 0.071	H-atom parameters constrained
*wR*(*F*^2^) = 0.199	*w* = 1/[σ^2^(*F*_o_^2^) + (0.0625*P*)^2^ + 1.8972*P*] where *P* = (*F*_o_^2^ + 2*F*_c_^2^)/3
*S* = 1.10	(Δ/σ)_max_ < 0.001
2557 reflections	Δρ_max_ = 0.43 e Å^−^^3^
155 parameters	Δρ_min_ = −0.33 e Å^−^^3^
0 restraints	Extinction correction: *SHELXL*, Fc^*^=kFc[1+0.001xFc^2^λ^3^/sin(2θ)]^-1/4^
Primary atom site location: structure-invariant direct methods	Extinction coefficient: 0.015 (3)

### Fractional atomic coordinates and isotropic or equivalent isotropic displacement parameters (Å^2^)

**Table:**

	*x*	*y*	*z*	*U*_iso_*/*U*_eq_	
C1	0.57261 (18)	−0.0804 (3)	0.3125 (4)	0.0320 (6)	
C2	0.6336 (2)	−0.1778 (3)	0.3751 (5)	0.0389 (7)	
H2	0.6170	−0.2628	0.3454	0.047*	
C3	0.7201 (2)	−0.1492 (3)	0.4825 (5)	0.0438 (7)	
H3	0.7618	−0.2149	0.5261	0.053*	
C4	0.7440 (2)	−0.0230 (3)	0.5246 (5)	0.0412 (7)	
H4	0.8020	−0.0027	0.5963	0.049*	
C5	0.6817 (2)	0.0725 (3)	0.4599 (5)	0.0401 (7)	
H5	0.6971	0.1582	0.4869	0.048*	
N1	0.59856 (15)	0.0413 (2)	0.3575 (4)	0.0342 (5)	
H1	0.5599	0.1021	0.3187	0.041*	
C6	0.47635 (19)	−0.1001 (3)	0.1946 (4)	0.0346 (6)	
C7	0.79780 (19)	0.0490 (3)	0.0421 (4)	0.0354 (6)	
C8	0.8827 (2)	−0.0055 (3)	0.1646 (5)	0.0373 (6)	
H8	0.8762	−0.0878	0.2130	0.045*	
C9	0.9664 (2)	0.0462 (3)	0.2149 (5)	0.0388 (7)	
H9	1.0084	−0.0061	0.2955	0.047*	
C10	1.0058 (2)	0.1714 (3)	0.1674 (5)	0.0401 (7)	
O1	0.45516 (17)	−0.2082 (2)	0.1321 (4)	0.0566 (7)	
O2	0.42766 (14)	0.0007 (2)	0.1721 (4)	0.0453 (6)	
H2A	0.3766	−0.0161	0.1075	0.068*	
O3	0.72935 (14)	−0.0300 (2)	0.0195 (4)	0.0446 (6)	
O4	0.79341 (16)	0.1568 (2)	−0.0311 (4)	0.0601 (8)	
O5	0.95136 (17)	0.2604 (2)	0.0741 (4)	0.0559 (7)	
H5A	0.8978	0.2340	0.0523	0.084*	
O6	1.08852 (16)	0.1899 (3)	0.2139 (4)	0.0580 (7)	

### Atomic displacement parameters (Å^2^)

**Table:**

	*U*^11^	*U*^22^	*U*^33^	*U*^12^	*U*^13^	*U*^23^
C1	0.0282 (13)	0.0299 (13)	0.0366 (14)	−0.0020 (10)	0.0024 (10)	0.0014 (11)
C2	0.0389 (15)	0.0262 (13)	0.0493 (17)	0.0000 (11)	0.0014 (13)	0.0055 (12)
C3	0.0374 (15)	0.0392 (16)	0.0514 (18)	0.0078 (13)	−0.0014 (13)	0.0081 (14)
C4	0.0284 (13)	0.0471 (17)	0.0440 (17)	−0.0014 (12)	−0.0051 (12)	−0.0031 (13)
C5	0.0310 (14)	0.0349 (15)	0.0518 (18)	−0.0029 (11)	0.0001 (12)	−0.0068 (13)
N1	0.0270 (11)	0.0274 (11)	0.0458 (14)	0.0014 (9)	−0.0005 (10)	0.0004 (10)
C6	0.0270 (13)	0.0327 (14)	0.0417 (15)	−0.0060 (10)	−0.0003 (11)	0.0041 (11)
C7	0.0295 (13)	0.0331 (14)	0.0426 (15)	0.0006 (11)	0.0031 (11)	−0.0015 (12)
C8	0.0358 (14)	0.0296 (13)	0.0451 (16)	0.0011 (11)	0.0029 (12)	0.0029 (12)
C9	0.0352 (14)	0.0320 (14)	0.0452 (17)	0.0020 (11)	−0.0041 (12)	0.0017 (12)
C10	0.0378 (15)	0.0350 (15)	0.0459 (17)	−0.0054 (12)	0.0024 (13)	−0.0057 (13)
O1	0.0479 (13)	0.0352 (12)	0.0781 (18)	−0.0090 (10)	−0.0125 (12)	−0.0019 (12)
O2	0.0261 (10)	0.0393 (12)	0.0648 (15)	−0.0008 (9)	−0.0079 (9)	−0.0009 (10)
O3	0.0323 (10)	0.0425 (12)	0.0555 (14)	−0.0055 (9)	−0.0018 (9)	0.0043 (10)
O4	0.0395 (12)	0.0409 (13)	0.092 (2)	−0.0009 (10)	−0.0120 (12)	0.0193 (13)
O5	0.0458 (13)	0.0373 (12)	0.0786 (18)	−0.0083 (10)	−0.0054 (12)	0.0109 (12)
O6	0.0378 (12)	0.0526 (15)	0.0787 (19)	−0.0125 (11)	−0.0037 (12)	−0.0083 (13)

### Geometric parameters (Å, º)

**Table:**

C1—N1	1.342 (4)	C6—O2	1.261 (3)
C1—C2	1.370 (4)	C7—O4	1.227 (4)
C1—C6	1.514 (4)	C7—O3	1.284 (3)
C2—C3	1.384 (4)	C7—C8	1.488 (4)
C2—H2	0.9300	C8—C9	1.328 (4)
C3—C4	1.376 (5)	C8—H8	0.9300
C3—H3	0.9300	C9—C10	1.484 (4)
C4—C5	1.369 (4)	C9—H9	0.9300
C4—H4	0.9300	C10—O6	1.213 (4)
C5—N1	1.336 (4)	C10—O5	1.315 (4)
C5—H5	0.9300	O2—H2A	0.8200
N1—H1	0.8600	O5—H5A	0.8200
C6—O1	1.224 (4)		
			
N1—C1—C2	118.8 (2)	O1—C6—O2	128.0 (3)
N1—C1—C6	116.8 (2)	O1—C6—C1	117.9 (3)
C2—C1—C6	124.4 (3)	O2—C6—C1	114.0 (2)
C1—C2—C3	119.7 (3)	O4—C7—O3	123.4 (3)
C1—C2—H2	120.1	O4—C7—C8	124.1 (3)
C3—C2—H2	120.1	O3—C7—C8	112.5 (3)
C4—C3—C2	119.5 (3)	C9—C8—C7	129.5 (3)
C4—C3—H3	120.2	C9—C8—H8	115.2
C2—C3—H3	120.2	C7—C8—H8	115.2
C5—C4—C3	119.5 (3)	C8—C9—C10	132.4 (3)
C5—C4—H4	120.3	C8—C9—H9	113.8
C3—C4—H4	120.3	C10—C9—H9	113.8
N1—C5—C4	119.4 (3)	O6—C10—O5	120.7 (3)
N1—C5—H5	120.3	O6—C10—C9	119.4 (3)
C4—C5—H5	120.3	O5—C10—C9	120.0 (3)
C5—N1—C1	123.1 (2)	C6—O2—H2A	109.5
C5—N1—H1	118.5	C10—O5—H5A	109.5
C1—N1—H1	118.5		
			
N1—C1—C2—C3	0.0 (5)	C2—C1—C6—O1	−8.5 (5)
C6—C1—C2—C3	−179.9 (3)	N1—C1—C6—O2	−7.8 (4)
C1—C2—C3—C4	−0.3 (5)	C2—C1—C6—O2	172.1 (3)
C2—C3—C4—C5	0.2 (5)	O4—C7—C8—C9	−1.2 (6)
C3—C4—C5—N1	0.2 (5)	O3—C7—C8—C9	178.2 (3)
C4—C5—N1—C1	−0.6 (5)	C7—C8—C9—C10	−1.2 (6)
C2—C1—N1—C5	0.5 (5)	C8—C9—C10—O6	−171.4 (4)
C6—C1—N1—C5	−179.7 (3)	C8—C9—C10—O5	8.0 (6)
N1—C1—C6—O1	171.6 (3)		

### Hydrogen-bond geometry (Å, º)

**Table:**

*D*—H···*A*	*D*—H	H···*A*	*D*···*A*	*D*—H···*A*
N1—H1···O2	0.86	2.28	2.639 (3)	105
O5—H5*A*···O4	0.82	1.73	2.540 (3)	168
N1—H1···O1^i^	0.86	2.02	2.725 (3)	139
O2—H2*A*···O3^ii^	0.82	1.71	2.463 (3)	152
C2—H2···O2^iii^	0.93	2.54	3.462 (4)	170
C3—H3···O6^iv^	0.93	2.59	3.221 (4)	125
C5—H5···O4^v^	0.93	2.40	3.251 (4)	152
C8—H8···O6^vi^	0.93	2.40	3.285 (4)	158

Symmetry codes: (i) −*x*+1, *y*+1/2, −*z*+1/2; (ii) −*x*+1, −*y*, −*z*; (iii) −*x*+1, *y*−1/2, −*z*+1/2; (iv) −*x*+2, −*y*, −*z*+1; (v) *x*, −*y*+1/2, *z*+1/2; (vi) −*x*+2, *y*−1/2, −*z*+1/2.
